# Author Correction: Directed self-assembly of herbal small molecules into sustained release hydrogels for treating neural inflammation

**DOI:** 10.1038/s41467-020-17712-5

**Published:** 2020-07-27

**Authors:** Jun Zheng, Rong Fan, Huiqiong Wu, Honghui Yao, Yujie Yan, Jiamiao Liu, Lu Ran, Zhifang Sun, Lunzhao Yi, Li Dang, Pingping Gan, Piao Zheng, Tilong Yang, Yi Zhang, Tao Tang, Yang Wang

**Affiliations:** 1 Key Laboratory of Hunan Province for Water Environment and Agriculture Product Safety, College of Chemistry and Chemical Engineering, Central South University, 410083 Changsha, China; 2 Institute of Integrative Medicine, Key Laboratory of Hunan Province for Liver Manifestation of Traditional Chinese Medicine, Xiangya Hospital, Central South University, 410008 Changsha, China; 3 Key Laboratry of Materials Processing and Mold, Ministry of Education, Zhengzhou University, 450002 Zhengzhou, China; 4 Xiangya School of Medicine, Central South University, 410013 Changsha, China; 5 Yunnan Food Safety Research Institute, Kunming University of Science and Technology, 650500 Kunming, China; 6 Department of Chemistry and Key Laboratory for Preparation and Application of Ordered Structural Materials of Guangdong Province, Shantou University, 515063 Shantou, China; 7 Department of Oncology, Xiangya Hospital, Central South University, 410008 Changsha, China; 8 College of Integrated Traditional Chinese and Western Medicine, Hunan University of Chinese Medicine, 410208 Changsha, China; 9 Southern University of Science and Technology, 518055 Shenzhen, China

**Keywords:** Biomedical materials, Drug delivery, Small molecules, Gels and hydrogels, Self-assembly

Correction to: *Nature Communications* 10.1038/s41467-019-09601-3, published online 8 April 2019.

The original version of this Article contained an error in Fig. 6b, in which the 48 h p-IkBα and NF-kB p65 panels looked identical. This has been corrected in both the PDF and HTML versions of the Article.

A correction to the supplementary information file has been made where the correct blots have replaced the incorrect bolts for Supplementary Fig. 13 for the 48 hour p-IkBα panel. These changes have been noted in the figure legend.

