# Supplementary material for: Directed self-assembly of herbal small molecules into sustained release hydrogels for treating neural inflammation
Source: Nat Commun. 2019 Apr 8;10:1604. doi: 10.1038/s41467-019-09601-3 (PMC6453967; doi:10.1038/s41467-019-09601-3)
Supplement: Supplementary file 1 — Supplementary Information [file 41467_2019_9601_MOESM1_ESM.pdf]

Supplementary Information

Direct self-assembly hydrogel of herbal small molecules towards a natural medicine

Jun Zheng et al.

Supplementary Figures

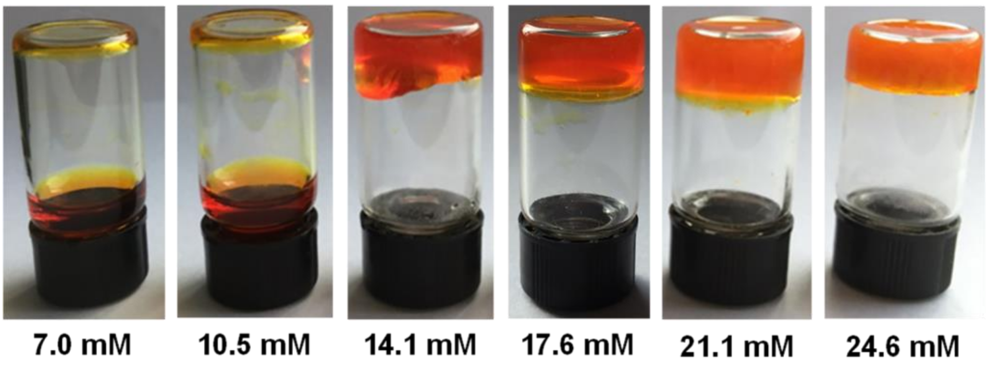

Supplementary Figure 1. Digital images of critical gelation concentration test.

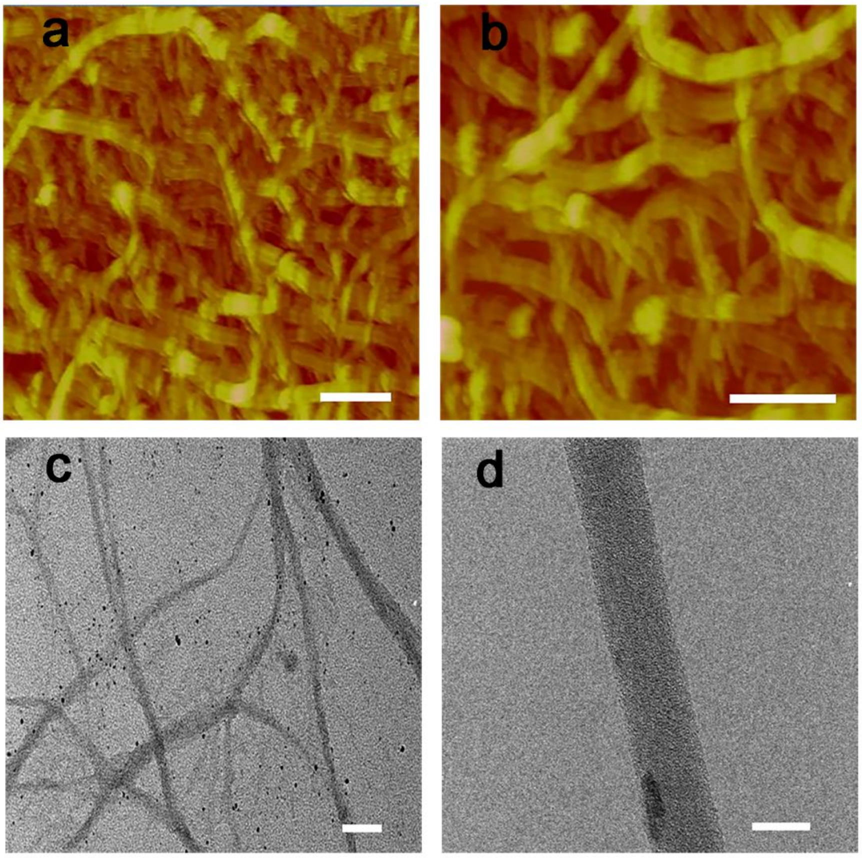

Supplementary Figure 2. Morphological characterization of the rhein hydrogel. (**a)** AFM image of the rhein hydrogel, scale bar, 500 nm. (**b**) Local amplification of a. scale bar: 500 nm. (**c**) TEM image of rhein hydrogel, scale bar: 100 nm. (**d**) Local amplification of c, scale bar: 20 nm.

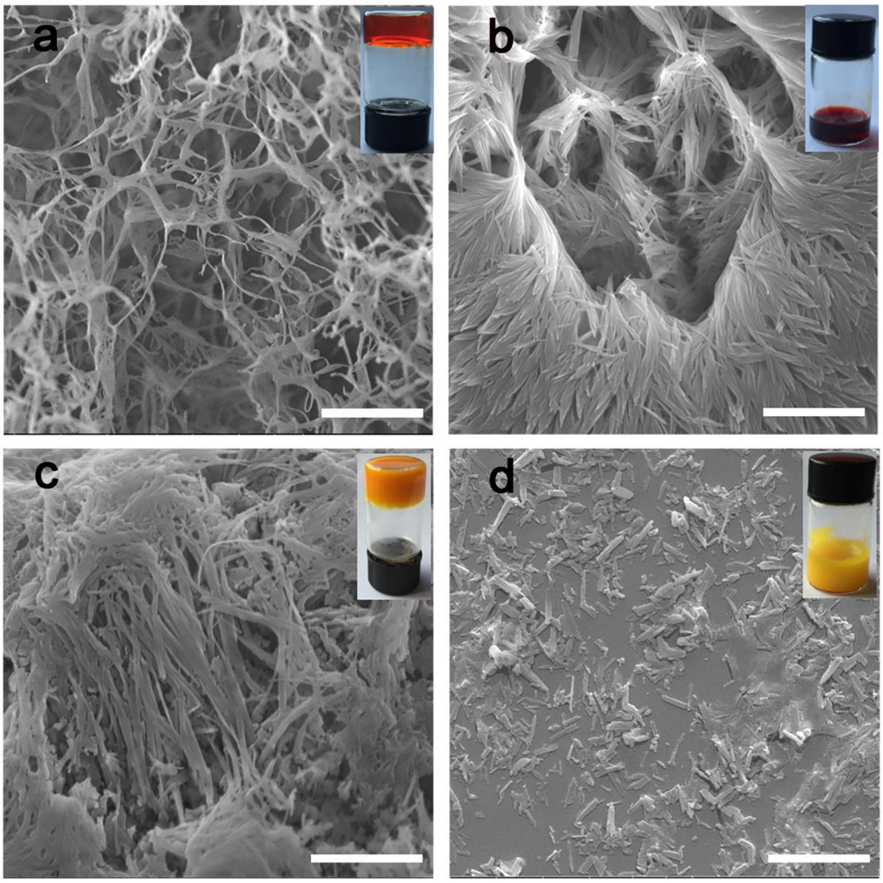

Supplementary Figure 3. SEM images of rhein at different pH values. (**a**) pH=8.3, (**b**) pH=10.2, (**c**) pH=7.4, (**d**) pH=6.5. All the rhein concentrations are 5 mg mL^{-1} (17.6 mM), scale bar: 5 μm.

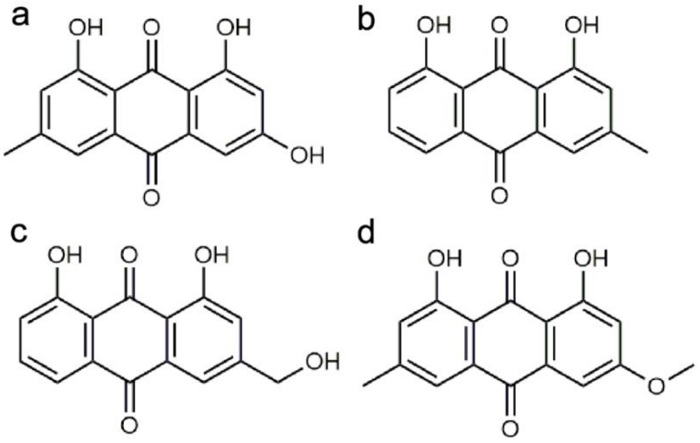

Supplementary Figure 4. The structural formula of (**a**) emodin, (**b**) chrysophanol, (**c**) aloe-emodin and (**d**) physcion.

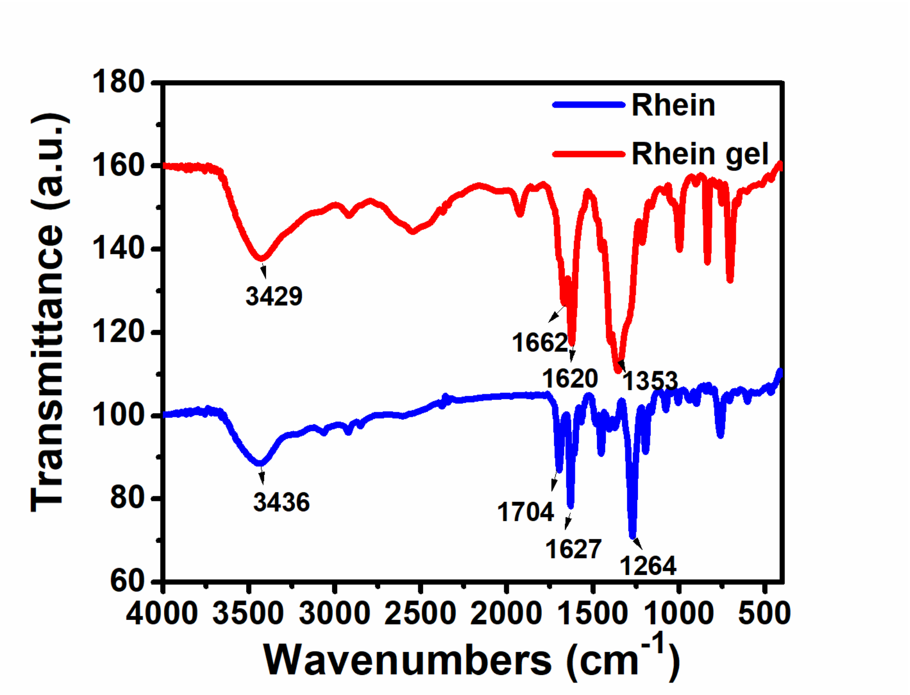

Supplementary Figure 5. FT-IR of the rhein and rhein hydrogel. Samples were prepared at a concentration of 4 mg mL^{-1}, scanned between 4000 and 400 cm^{-1}.

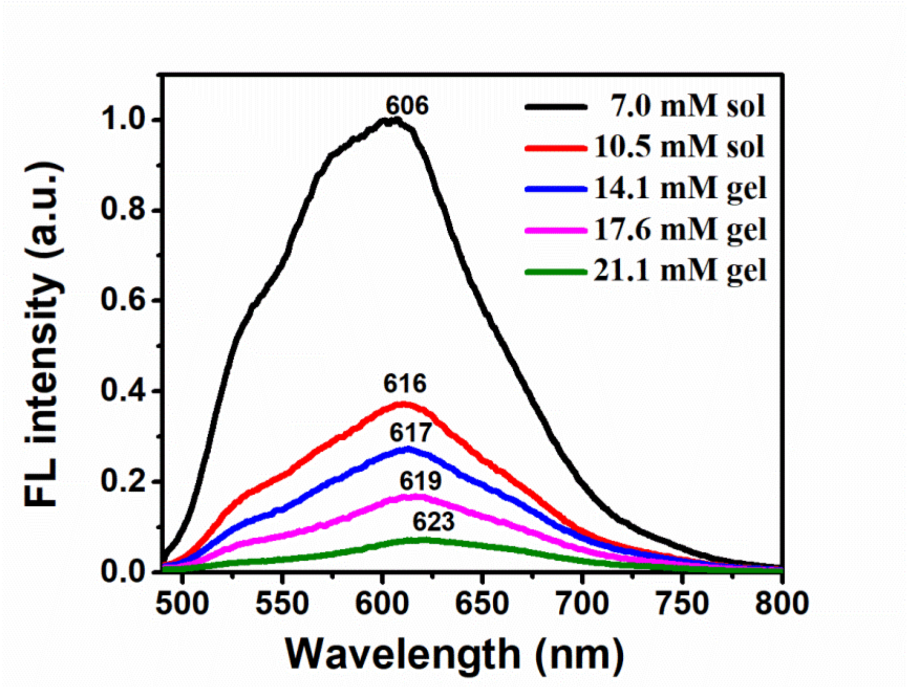

Supplementary Figure 6. The fluorescence emission spectrum of the rhein hydrogel.

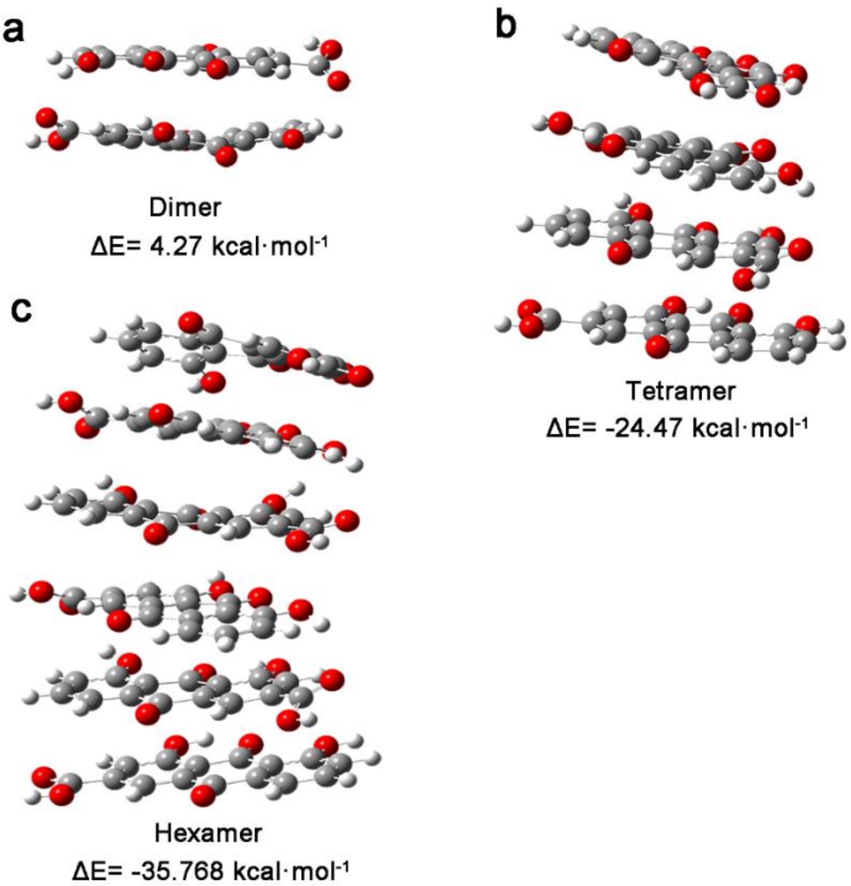

Supplementary Figure 7. Configuration and energy value of the rhein aggregates. (**a**) Dimer, (**b**) Tetramer and (**c**) Hexamer, respectively.

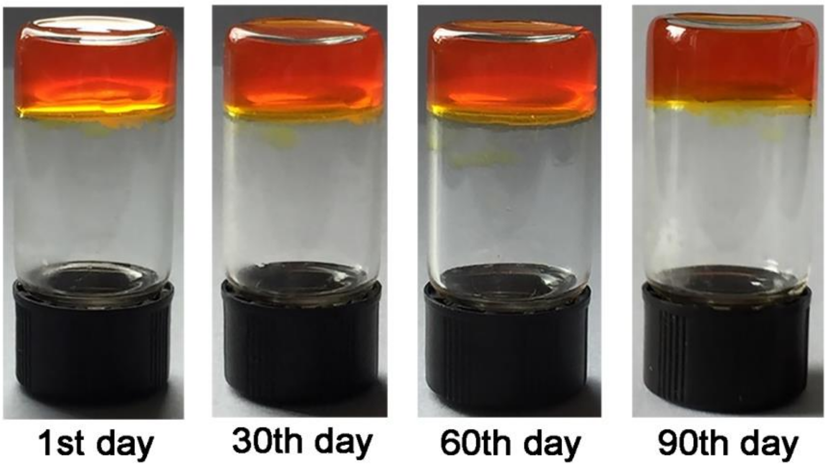

Supplementary Figure 8. The digital photos of rhein hydrogel at different time points. The concentration of hydrogel was 4 mg mL^{-1} (17.6 mM).

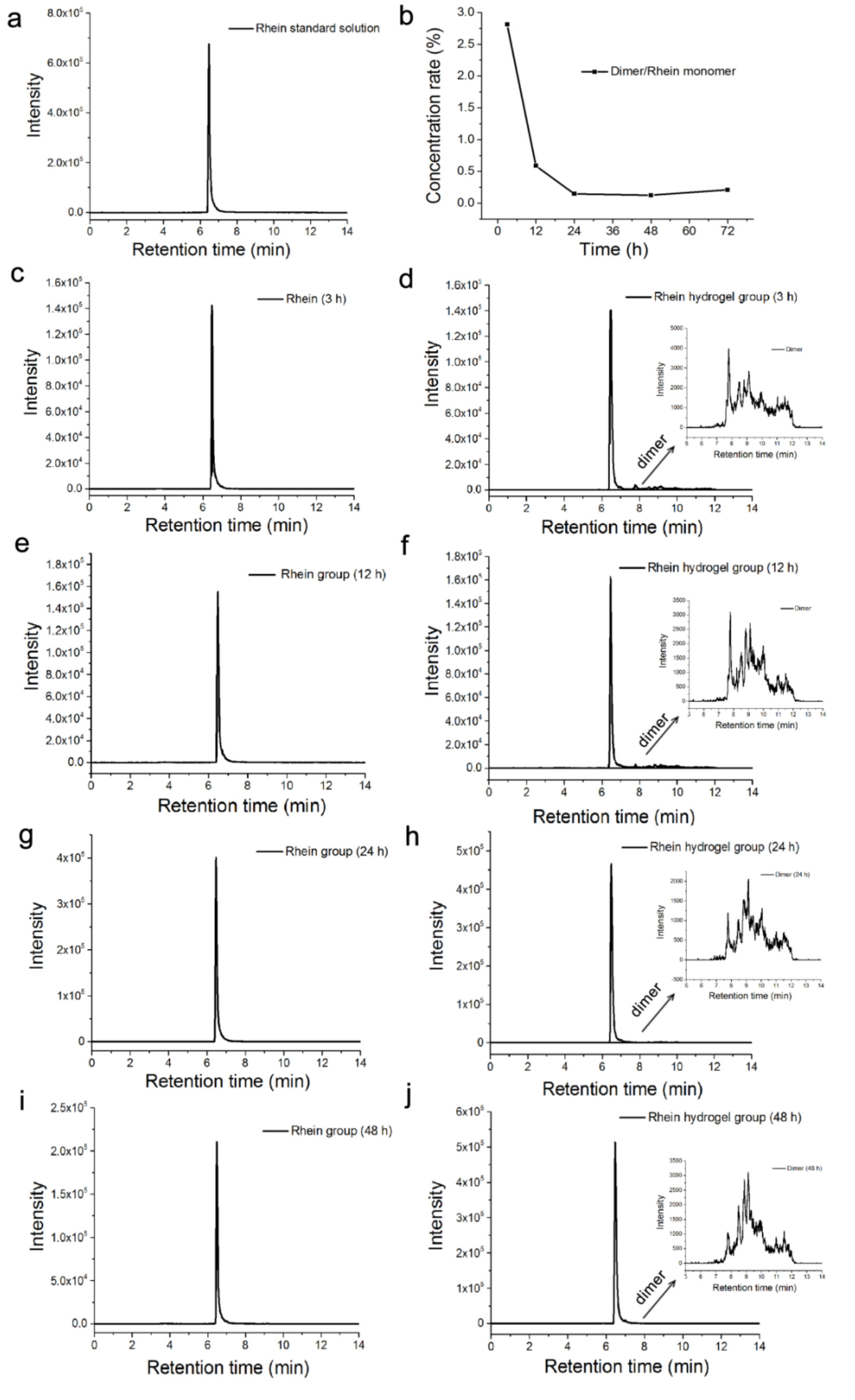

Supplementary Figure 9. LC-MS analysis of rhein and dimer in BV2 cells. (**a**) Rhein standard solution (**b**) Concentration ratio of dimer to rhein monomer in cells cultured with hydrogel. (**c**) Rhein group at 3 h. (**d**) Rhein hydrogel group at 3 h. (**e**) Rhein group at 12 h, (**f**) Rhein hydrogel group at 12 h, (**g**) Rhein group at 24 h. (**h**) Rhein hydrogel group at 24 h. (**i**) Rhein group at 48 h. (**j**) Rhein hydrogel group at 48 h.

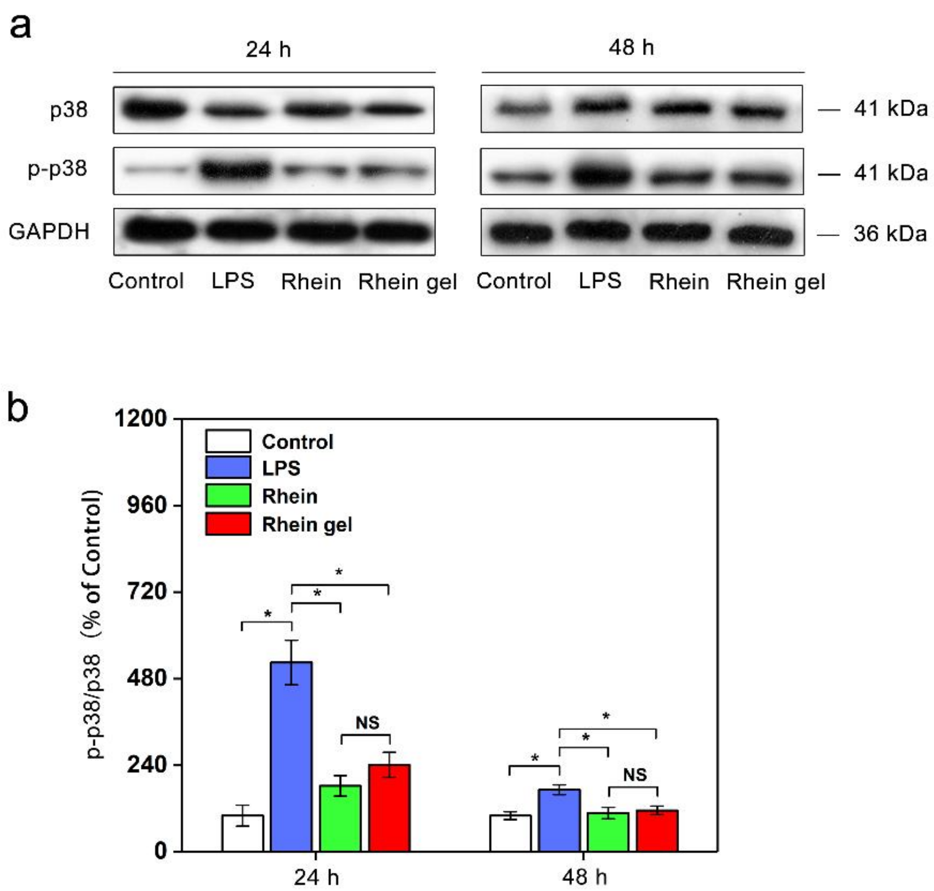

Supplementary Figure 10. Rhein and its hydrogel blocked p38 pathway. (**a**) Representative western blots of p38 after treatment of rhein and its hydrogel for 24 h and 48 h. (**b**) Quantification of p38 according to densitometric analysis. Values are expressed as the means ± SEM (n = 3). One-way ANOVA with Tukey's post hoc test. *p < 0.05. Error bars represent SEM.

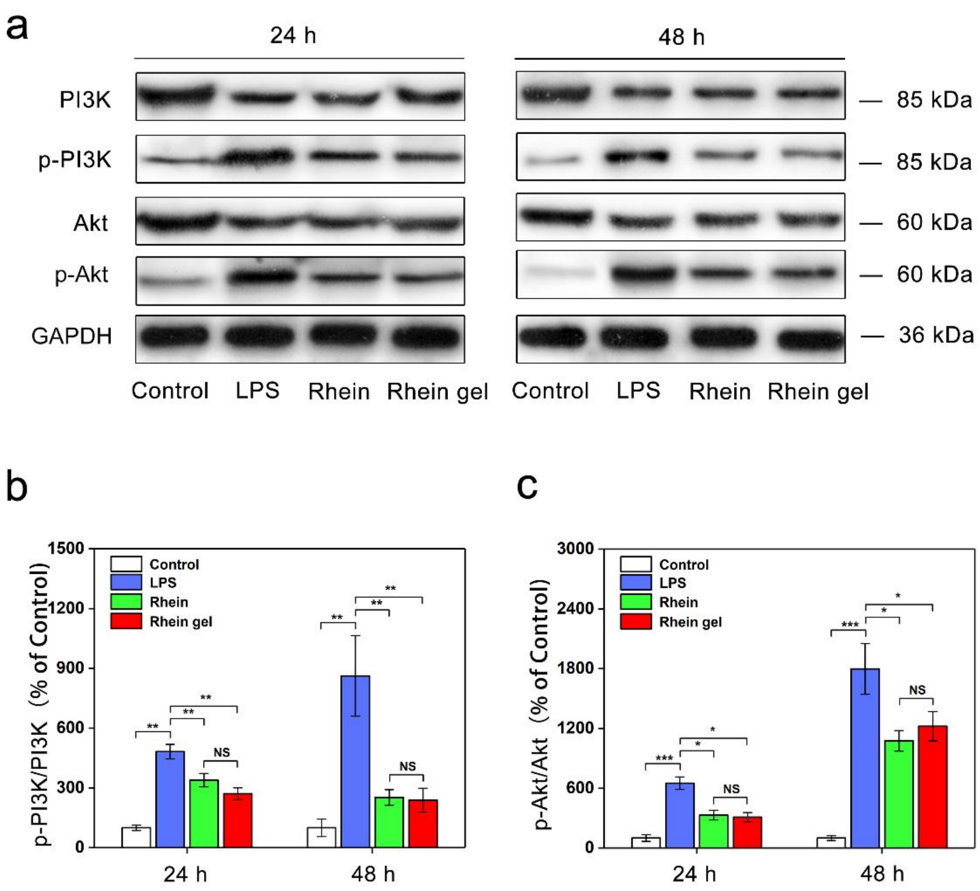

Supplementary Figure 11. Rhein and its hydrogel blocked PI3K/Akt pathway. (**a**) Representative western blots of PI3K, p- PI3K, Akt and p-Akt after treatment of rhein or rhein hydrogel for 24h and 48h. (**b**, **c**), Quantification of p-PI3K/PI3K (**b**) and p-Akt/Akt (**c**) according to densitometric analysis. Values are expressed as the means ± SEM (n = 3). One-way ANOVA with Tukey's post hoc test. *p < 0.05, **p < 0.01, ***p < 0.001. Error bars represent SEM.

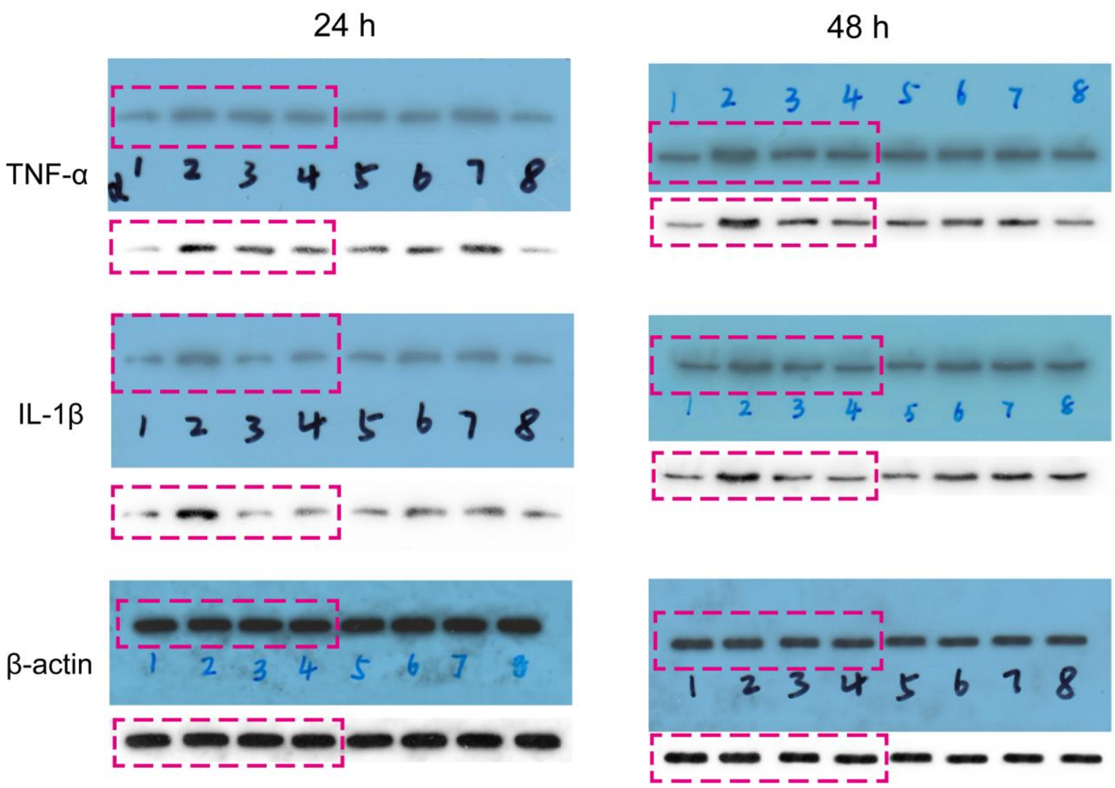

Supplementary Figure 12. Uncropped blots scans of all blots in Fig. 5b.

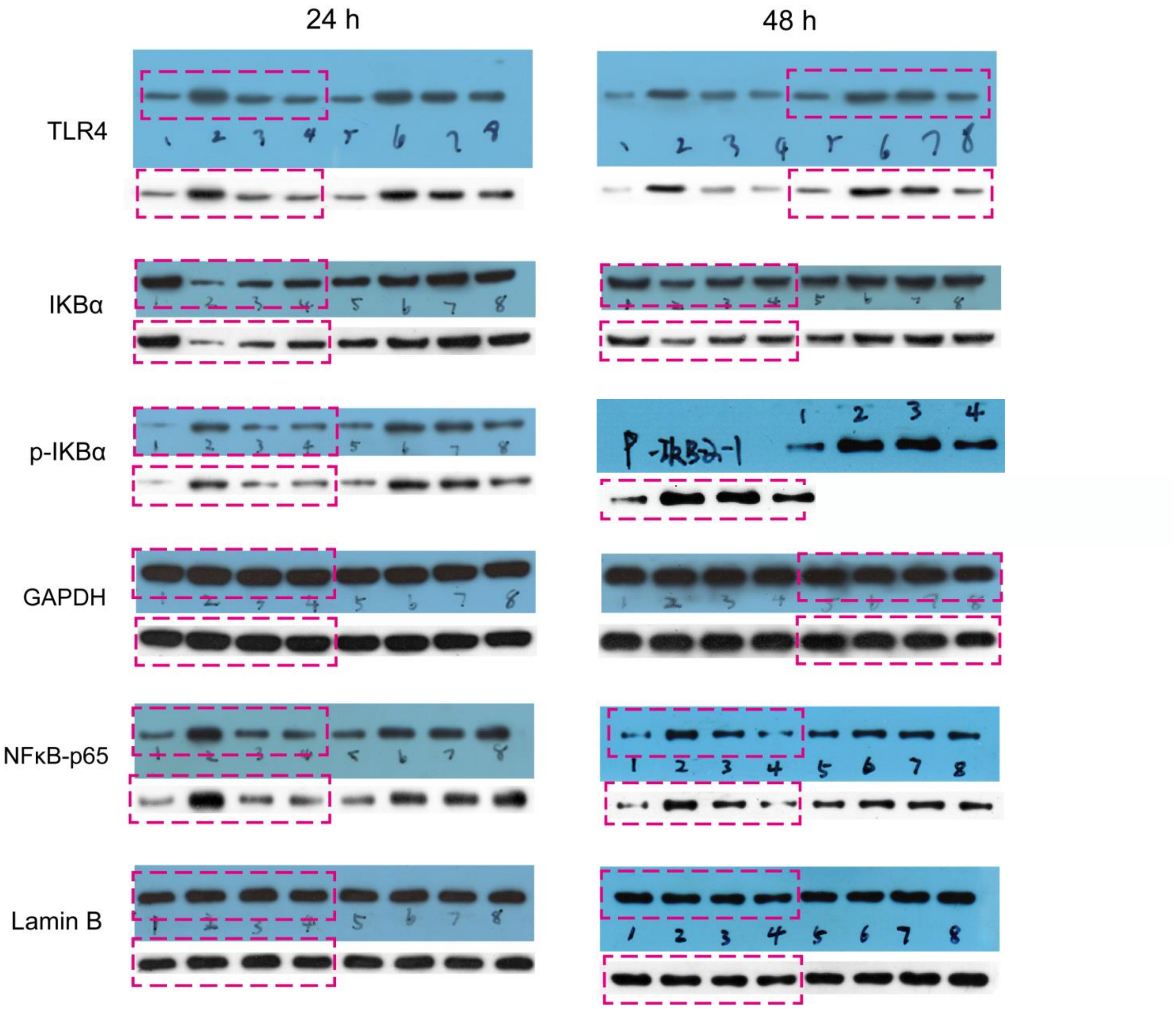

Supplementary Figure 13. Uncropped blots scans of all blots in Fig. 6b (Correction; 48h p-IkBa has been replaced with correct blots at authors request).
